# Beyond anaerobic respiration—new physiological roles for DmsABC and other S-/N-oxide reductases in *Escherichia coli*

**DOI:** 10.1128/jb.00463-24

**Published:** 2025-03-31

**Authors:** Qifeng Zhong, Marufa Nasreen, Ruizhe Yang, Michel Struwe, Bostjan Kobe, Ulrike Kappler

**Affiliations:** 1 School of Chemistry and Molecular Biosciences, The University of Queensland 198110 , St. Lucia, Queensland, Australia; 2 Institute for Molecular Bioscience, The University of Queensland 85088, St. Lucia, Queensland, Australia; University of Virginia School of Medicine, Charlottesville, Virginia, USA

**Keywords:** sulfoxide reductases, molybdenum enzymes, oxidative stress, uropathogenic *Escherichia coli*, bacterial virulence

## Abstract

Sulfoxide reductases in pathogenic bacteria have recently received increasing attention for their association with virulence and survival within the host. Here, we have re-investigated the physiological role of the molybdenum-containing DmsABC dimethyl sulfoxide (DMSO) reductase from *Escherichia coli*, which has a proposed role in anaerobic respiration with DMSO. Our investigation into potential physiological substrates revealed that DmsABC efficiently reduces pyrimidine N-oxide, nicotinamide N-oxide, and methionine sulfoxide, and exposure to host cell-produced stressors such as hypochlorite or hydrogen peroxide specifically increased expression of the *E. coli dmsA* gene. *E. coli* strains lacking *dmsA* showed increased lag times in the presence of hypochlorite, and these strains also showed up to a 90% reduction in adherence to human bladder cells. Interestingly, in the presence of hypochlorite, expression of multiple alternative S-/N-oxide reductases present in *E. coli* was elevated by 2- to 4-fold in a ∆*dmsA* strain compared to the wild-type strain, suggesting functional redundancy. The phenotypes of the *E. coli* ∆*dmsA* strains were strikingly similar to ∆*dmsA* strains of the respiratory pathogen *Haemophilus influenzae*, which confirms the role of both enzymes in supporting host-pathogen interactions. We propose that this function is conserved in enzymes closely related to *E. coli* DmsABC. Our study also uncovered that the expression of many *E. coli* Mo enzymes was induced by oxidative stressors, including metals such as copper, and further work should be directed at determining the connection of these enzymes to host-pathogen interactions.

**IMPORTANCE:**

Bacterial urinary tract infections are debilitating and frequently recurring in human populations worldwide, and *Escherichia coli* strains are a major cause of these infections. In this study, we have uncovered a new mechanism by which *E. coli* can avoid being killed by the human immune system. The bacteria use a set of seven related enzymes that can reverse damage to essential cell components such as amino acids, vitamins, and DNA building blocks. Antibacterial compounds produced by the human immune system specifically induced the production of these enzymes, confirming that they play a role in helping *E. coli* survive during infection and making these enzymes potential future drug targets.

## INTRODUCTION

**Fig 1:**
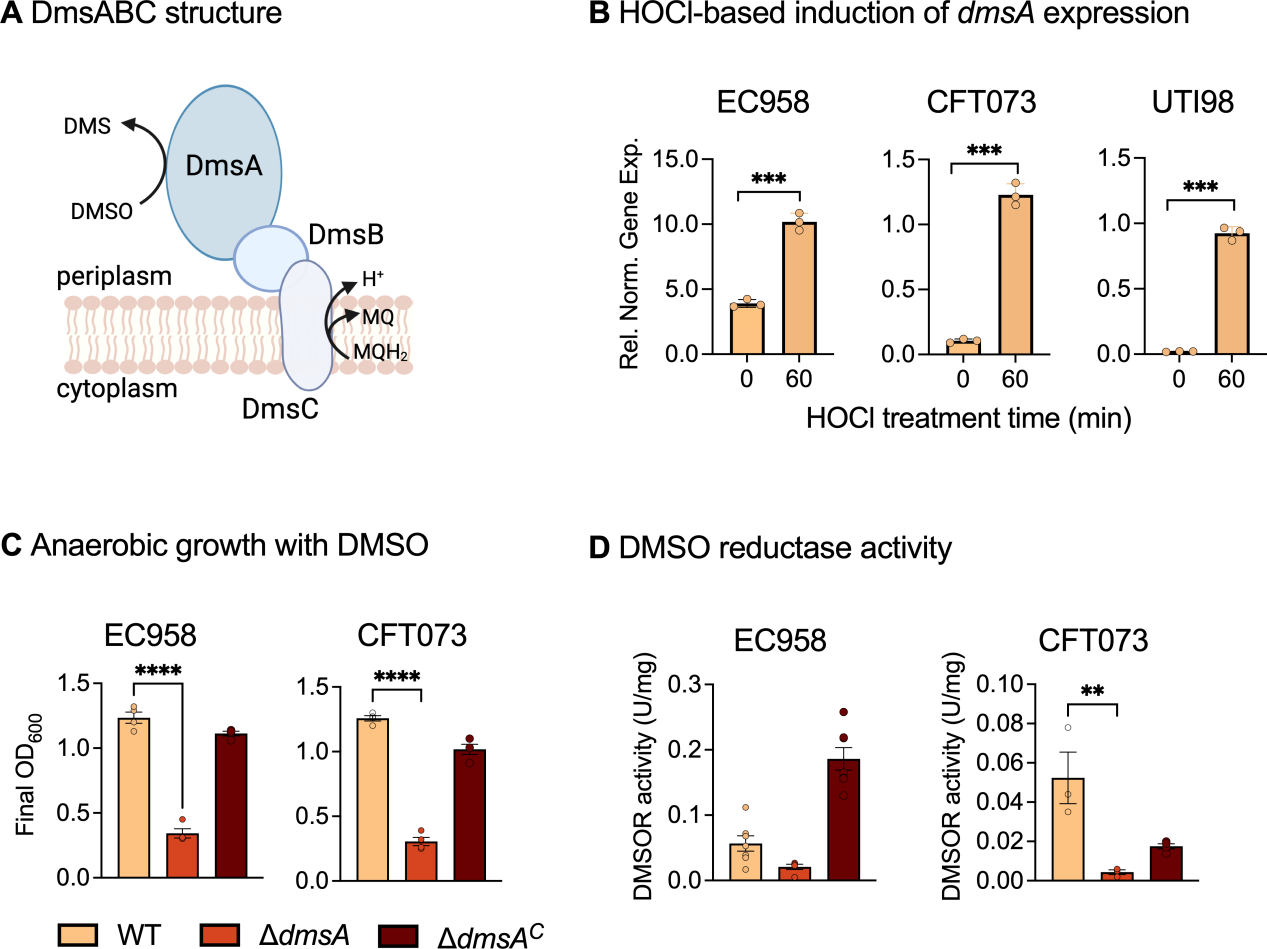
*E*. *coli* DmsABC expression in response to oxidative stress and characterization of ∆*dmsA* strains. Panel (A): Schematic representation of the *E. coli* DmsABC enzyme complex. DMS, dimethyl sulfide; DMSO, dimethyl sulfoxide; MQ, menaquinone; MQH_2_, menaquinol. Panel (B): Expression *dmsA* in the EC958 WT, CFT073 WT, and UTI89 WT strains following a mid-log-phase HOCl challenge under microaerobic conditions. Data were normalized using *gyrA* gene expression. Data points represent technical replicates, and error bars represent the standard error of the mean. Two-tailed unpaired *t*-tests were performed for statistical comparison: ***P < 0.001. Panel (C): Comparison of the ability of the UPEC EC958 and CFT073 WT, ∆*dmsA,* and ∆*dmsA^C^* strains to respire anaerobically with DMSO. For DMSO-based respiration, the bacterial strains were cultured anaerobically in glycerol-DMSO medium for 40 h, at which point the OD_600_ values of the cultures were recorded; data points represent biological replicates. Panel (D) : DMSO reductase activities in cell extracts of UPEC EC958 and CFT073 WT, ∆*dmsA,* and ∆*dmsA^C^* strains. Cell extracts were from cultures grown anaerobically on glycerol-fumarate medium for 40 h; data points represent technical replicates of a representative experiment. Error bars represent the standard error of the mean. Statistical analyses used one-way ANOVA with Tukey’s multiple comparison correction. *P ≤ 0.05; **P ≤ 0.01; ***P ≤0.001; and ****P ≤ 0.0001. ANOVA, analysis of variance; HOCl, hypochlorite; WT, wild-type.

Mononuclear molybdenum (Mo) enzymes were discovered nearly a century ago in 1924, but only recently has their importance for bacterial fitness and virulence become a focus (1–3). Various recent studies have linked in-host survival of prevalent pathogenic bacteria such as *Mycobacterium tuberculosis*, *Escherichia coli*, *Haemophilus influenzae*, and *Salmonella enterica* to the presence of functional Mo enzymes (1, 2, 4–7). The DmsABC dimethyl sulfoxide (DMSO) reductase (DMSOR) is a member of the DMSOR family of mononuclear Mo enzymes and has been extensively studied in *E. coli* following its discovery in the late 1980s (8–11). DmsABC has been proposed to reduce DMSO to dimethyl sulfide during anaerobic (AN) energy generation. It comprises a periplasmic, catalytic subunit, DmsA, which contains a Mo-bisPGD cofactor and a 4Fe-4S cluster (Fig. 1A), an electron-transferring subunit, DmsB, with four 4Fe-4S clusters, and a membrane-anchoring subunit, DmsC, that connects the enzyme to the cellular quinone-pool as a source of electrons (11–14). DmsA and DmsB are exported to the periplasm via the twin-arginine translocation (Tat) pathway, a process that requires the DmsA Tat signal peptide and a chaperone protein, DmsD (14–16). Despite being essential for DmsABC maturation, in *E. coli*, DmsD is encoded by a gene located downstream of the *ynfEFGH* genes that encode putative selenate reductase that is a paralogue of DmsABC (17). By contrast, in the human respiratory pathogen *H. influenzae*, where the function of DmsABC has recently been investigated, the operon that encodes *dmsABC* includes two genes for chaperone proteins, *dmsD* and *dmsE* (5, 18), demonstrating that the *dmsABC* operon structure is not evolutionarily conserved.

Functional and physiological studies have led to the proposal that in *E. coli*, DmsABC supports AN respiration with DMSO, as strains carrying a mutation in *dmsA* were unable to grow anaerobically by DMSO respiration (13). The expression of *dmsA* was found to be controlled by the transcription factors ModE and FNR in response to the presence of molybdate and under AN conditions, respectively (19, 20). However, both ModE and FNR are general regulators that control a variety of genes and are not specific for *dmsA* expression (21, 22). Interestingly, the presence of the putative natural substrate, DMSO, does not induce the expression of *dmsA* in *E. coli* (19), and DMSO is only present in minimal concentrations in the human gut (23), where *E. coli* primarily resides (24). By contrast, the expression of DMSORs from bacteria that inhabit environments where DMSO occurs naturally, such as the DorA DMSOR from *Rhodobacter* spp., increases strongly in the presence of DMSO (23, 25, 26).

Recently, a new role for DmsABC has been identified in the human respiratory pathogen *H. influenzae*, where the enzyme is required for survival during infection (5, 18). In a murine lung infection model, the *H. influenzae* ∆*dmsA* strain showed a severe survival defect, with the number of viable bacteria dropping to less than 0.01% of the wild-type (WT) strain by day 2 of infection (5). Similar observations were also made for infections of primary human nasal epithelia, where the mutant strains were outcompeted by the WT (18). Similar results have been reported for the pig pathogen *Actinobacillus pleuropneumoniae*, where a ∆*dmsA* strain was unable to induce acute pleuropneumonia (27), and in *S. enterica* serotype Typhimurium, where mouse gut colonization was disrupted in the absence of DmsABC-like sulfoxide reductases (4).

DmsABC from *H. influenzae* and *E. coli* are closely related enzymes (DmsA amino acid sequence identity: 73%), and the expression of both is induced by anaerobiosis, but not the presence of DMSO (5, 19, 28). In keeping with the role of DmsABC in in-host survival, hypochlorite (HOCl) and superoxide, which are produced by the host during infection, specifically induced *dmsA* expression in *H. influenzae* (18). HOCl is a product of the host enzyme myeloperoxidase and has been estimated to reach local concentrations of up to 25 mM, while *in vitro* 0.1 µM HOCl was produced by activated neutrophils (29, 30). HOCl and superoxide cause oxidative damage predominantly to sulfur-containing biomolecules such as methionine and cysteine, rendering them biologically inactive (31–35). *H. influenzae* DmsABC (HiDmsABC) re-reduces methionine sulfoxide (MetSO), nicotinamide N-oxide (NNO), and pyrimidine N-oxide (PNO) with *K*_M_ values in the low micromolar range (18), reversing oxidative damage caused by HOCl and related compounds (5).

Given the similarities between *H. influenzae* and *E. coli* DmsABC, we have re-investigated the physiological role of EcDmsABC, including whether this enzyme also supports host-pathogen interactions rather than primarily mediating AN respiration with DMSO. Using two strains of uropathogenic *E. coli* (UPEC), we were able to demonstrate that the expression of *dmsA* was HOCl-inducible. The presence of HOCl reduced the growth of ∆*dmsA* strains *in vitro*, and during infection of bladder tissue cells, UPEC ∆*dmsA* strains showed reduced tissue cell adherence. Further experiments revealed that the expression of several alternative S- and N-oxide reductases present in *E. coli* was increased in the ∆*dmsA* strain compared to the WT strain, suggesting that these enzymes may be able to functionally compensate for the absence of DmsABC during infection.

## RESULTS

### Expression of *dmsA* in *E. coli* UPEC strains is induced by HOCl

UPEC is one of the most prominent groups of pathogenic *E. coli*. UPEC accounts for more than 80% of urinary tract infections (UTIs), with over 150 million individuals infected globally each year, causing $1.6 billion per annum in medical expenses in the the United States alone (36, 37). In Australia, UTIs were the third most common healthcare-associated infection in 2022 (38). Genes encoding DmsABC are conserved in *E. coli* genomes, and to study the link between EcDmsABC and host-pathogen interactions, we initially determined the effect of HOCl on *dmsA* expression in various UPEC strains, namely the multidrug-resistant UTI isolate EC958 (ST131), the pyelonephritis isolate CFT073 (ST73), and the cystitis isolate UTI89 (ST95) (39–41). In LB medium, exposure of mid-log phase microaerobic (MA) cultures of the three UPEC strains to HOCl for 60 min increased expression of *dmsA* 4.6, 11, and ~47 times (*t*-test, *P* < 0.0001, Fig. 1B), respectively, when compared to the corresponding untreated cultures. In M9-glucose minimal medium, *dmsA* expression in EC958 peaked at 15 min exposure to 50 µM HOCl before decreasing to roughly double the pre-induction levels for the remainder of the experiment (Fig. S1).

These experiments clearly demonstrate that HOCl, a product of the human immune system, specifically induces *dmsA* expression in *E. coli* growing on either minimal or rich growth media, similar to what we previously demonstrated for *H. influenzae* (18).

### UPEC *∆dmsA* strains showed the expected inability to grow by AN respiration with DMSO as the electron acceptor

To explore the physiological role of EcDmsABC, we constructed ∆*dmsA* mutant strains in UPEC strains EC958 and CFT073 using lambda Red-mediated recombination (42). The mutations were complemented using the native *E. coli dms* promoter and *dmsABC* genes cloned into the pSU2718-G vector (43). Mutations in *dmsA* have been constructed previously in other *E. coli* strains (13), and these strains were unable to grow by AN respiration with DMSO. We therefore grew the EC958 and CFT073 ∆*dmsA* strains on glycerol-DMSO minimal medium, where glycerol is the only and non-fermentable carbon source, and DMSO is the only electron acceptor present to validate the mutant strains (44). After 40 h of growth on this medium, the EC958 and CFT073 ∆*dmsA* strains only reached 24% and 28% of the WT level final OD_600_, respectively (Fig. 1C), indicating that DMSO-based respiration was impaired in the mutant strains. Complementation restored the growth of the EC958 and CFT073 mutant strains to greater than 90% of WT levels (Fig. 1C) (13).

We then tested DMSOR activity in cell extracts of the UPEC strains. Following AN growth, the EC958 and CFT073 ∆*dmsA* strains exhibited 37% and 8% of the WT strains’ DMSOR activity, respectively (Fig. 1D). Complementation restored activity; however, in the complemented EC958 ∆*dmsA^C^* strain, DMSOR activity exceeded that of the WT strain, while in CFT073, complementation only restored ~35% of WT level activity. As the *dmsABC* gene regions in these two strains share 97.6% nucleotide sequence identity, it is unclear why such strong differences in complementation levels were observed. However, even the reduced restoration of activity in the CFT073 strain was sufficient to restore growth on glycerol/DMSO medium.

### UPEC ∆*dmsA* strains exhibited strain-dependent growth defects following exposure to HOCl

**Fig 2:**
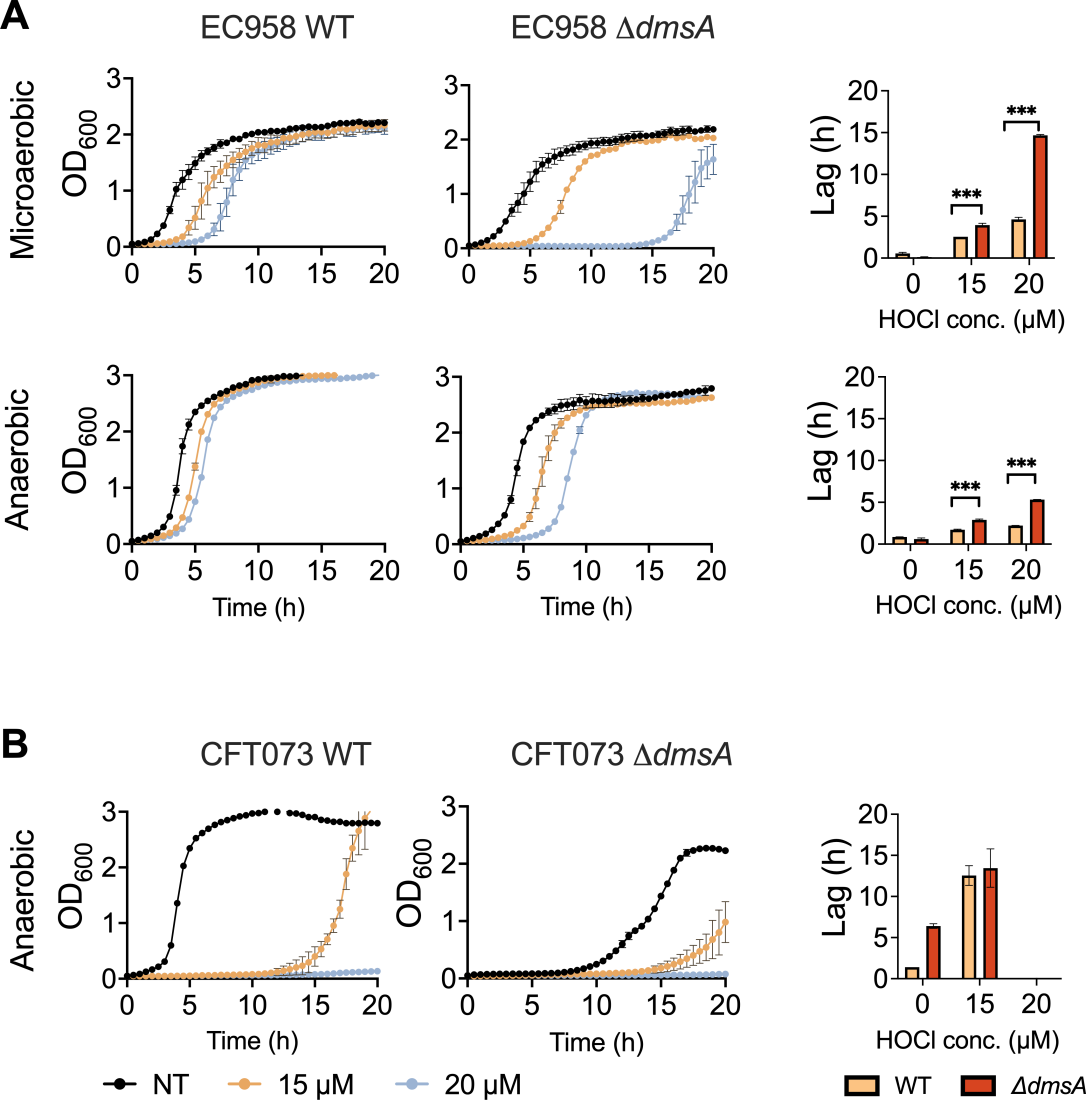
HOCl sensitivity of UPEC EC958 and CFT073 strains. Panel (A): Growth of EC958 WT and ∆*dmsA* strains in the presence of HOCl in M9-glucose medium under anaerobic or microaerobic conditions. Lag times are shown as a bar graph. Panel (B): Growth of CFT073 WT and ∆*dmsA* strains in the presence of HOCl in M9-glucose medium under anaerobic or microaerobic conditions. CFT073 strains did not grow in the presence of 20 µM HOCl. Lag times are shown as a bar graph. Representative data of three independent experiments with two biological replicates each are shown; error bars represent the standard error of the mean. Two-way ANOVA with Tukey’s multiple comparison correction to compare growth lag of the WT and mutant strains, with or without treatment. ***P ≤ 0.001; ****P <≤ 0.0001. ANOVA, analysis of variance; HOCl, hypochlorite; UPEC, uropathogenic *Escherichia coli*; WT, wild-type.

As HOCl induces expression of *dmsA* in *E. coli*, we then assessed the effect of HOCl stress on the ∆*dmsA* strain growth on M9-glucose medium. Under AN conditions, lag times for the EC958 WT strain increased by 1 and 1.5 h following treatment with 15 and 20 µM HOCl, respectively, while lag times for the EC958 ∆*dmsA* strain increased to 2 and 5 h under the same conditions (Fig. 2A). The effect of HOCl exposure was even more pronounced under MA conditions (Fig. 2A), where lag times of 2 and 4 h for the WT and 4 and 15 h for the ∆*dmsA* strain were observed, respectively, at the same HOCl concentrations. Despite the significant differences in lag times, the growth rates of the EC958 WT and ∆*dmsA* strains differed by less than 12%, regardless of oxygen tension or HOCl concentration (Table S1). Interestingly, for the CFT073 WT and ∆*dmsA* strains, the effect of HOCl treatment was markedly stronger already under AN conditions. Lag times of 12.5 and 14 h, respectively, were observed for the two strains following treatment with 15 µM HOCl (Fig. 2B). Interestingly, the growth rates of the CFT073 WT and ∆*dmsA* strains differed by ~63%, and the presence of HOCl reduced the growth rate of the WT strain by 33% (Table S1). The presence of 20 µM HOCl inhibited the growth of both CFT073 strains.

In summary, the *E. coli* ∆*dmsA* strains were more susceptible to HOCl-induced damage as evidenced by the increased lag times, and unexpectedly, as DmsABC has been associated mostly with AN growth of *E. coli*, this effect became more pronounced during MA growth, that is, under conditions where we also observed induction of *dmsA* expression in the presence of HOCl. This indicates a connection between DmsA and oxidative processes taking place during growth in the presence of oxygen.

### Loss of DmsA increased resistance to HOCl killing in EC958 but not CFT073

**Fig 3:**
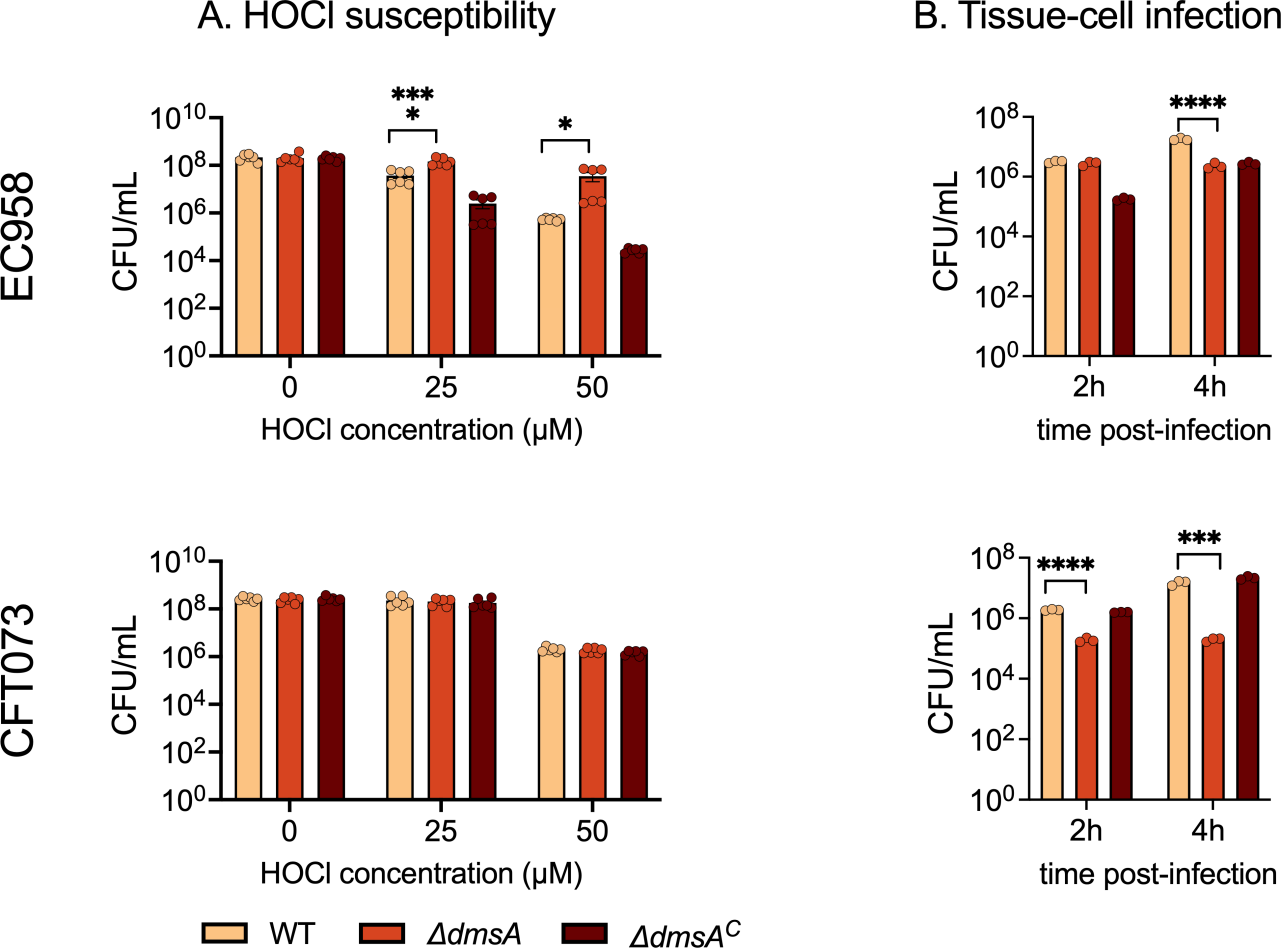
A loss of *dmsA* alters hypochlorite (HOCl) stress resistance and reduces survival of UPEC strains in tissue cell infections. Panel (A): Survival of the *E. coli* UPEC EC958 and CFT073 WT, ∆*dmsA,* and ∆*dmsA^C^* strains following exposure to increasing concentrations of HOCl for 60 min. Data from two independent experiments with three biological replicates each are shown. Panel (B): Adherent *E. coli* UPEC EC958 and CFT073 WT, ∆*dmsA,* and ∆*dmsA^C^* strains recovered after 2 and 4 h infection time from T24 human bladder epithelial cells. Data points represent biological replicates, and error bars represent the standard error of the mean. One-way ANOVA was performed with Tukey’s multiple comparison correction, statistical significance against the WT is shown as *P ≤ 0.05; ***P ≤ 0.0003; and ****P ≤ 0.0001. ANOVA, analysis of variance; WT, wild-type; UPEC, uropathogenic *Escherichia coli*.

As HOCl had a strong effect on the growth of the *E. coli* ∆*dmsA* strains, we next tested the susceptibility of non-growing EC958 and CFT073 WT and ∆*dmsA* strains to HOCl treatment by exposing ~2 × 10^8^ bacteria to 25 or 50 µM HOCl for 60 min. CFT073 WT and ∆*dmsA* strains showed similar sensitivity to HOCl. However, the EC958 ∆*dmsA* strain was less affected by HOCl treatment than the EC958 WT strain (Fig. 3A). Exposure to 25 µM HOCl reduced the viable bacterial counts of the EC958 WT strain by an order of magnitude, while that of the ∆*dmsA* strain was only reduced 1.3-fold. At 50 µM HOCl, viable bacterial counts of the EC958 ∆*dmsA* strain were over 60 times higher compared to the WT and ∆*dmsA^C^* strains (Fig. 3A). While the responses of the two *E. coli* strains to HOCl exposure differed, the EC958 ∆*dmsA* phenotype is consistent with the phenotype of an *H. influenzae* ∆*dmsA* strain (5), where a two-order-of-magnitude increase in viable bacterial counts was observed for the ∆*dmsA* strain following treatment with 0.2 mM HOCl. Despite the consistency in phenotype of ∆*dmsA* strains in two different species of bacteria, the cause of this unexpected resistance of some ∆*dmsA* strains to HOCl killing is unclear. A possible explanation could be that the loss of DmsABC leads to a change in the composition and function of the *E. coli* respiratory chain, which in turn could increase resistance to some antimicrobials, as reviewed in reference 45.

### The DmsABC substrate spectrum extends beyond DMSO and includes several biologically relevant S- and N-oxides

As DMSO is generally absent in body niches that *E. coli* strains inhabit and EcDmsABC appears to play a physiological role during HOCl exposure, EcDmsABC might use oxidatively damaged biomolecules such as amino acids, vitamins, or nucleobases as its main substrates. EcDmsABC has previously been shown to convert over 20 different S- and N-oxides (11, 46); however, most of the substrates tested do not occur in biological systems. In addition to DMSO, we selected MetSO, biotin sulfoxide (BSO), NNO, and PNO as representative oxidatively damaged biomolecules that could occur in the host environment during infection and be relevant for *E. coli* cell metabolism. As *E. coli* contains a range of other S- and N-oxide reductases (1), we conducted assays with cell extracts of the WT and the ∆*dmsA* strains to determine both the overall activity with a substrate and the DmsABC-independent activity levels with each substrate, respectively.

**Fig 4:**
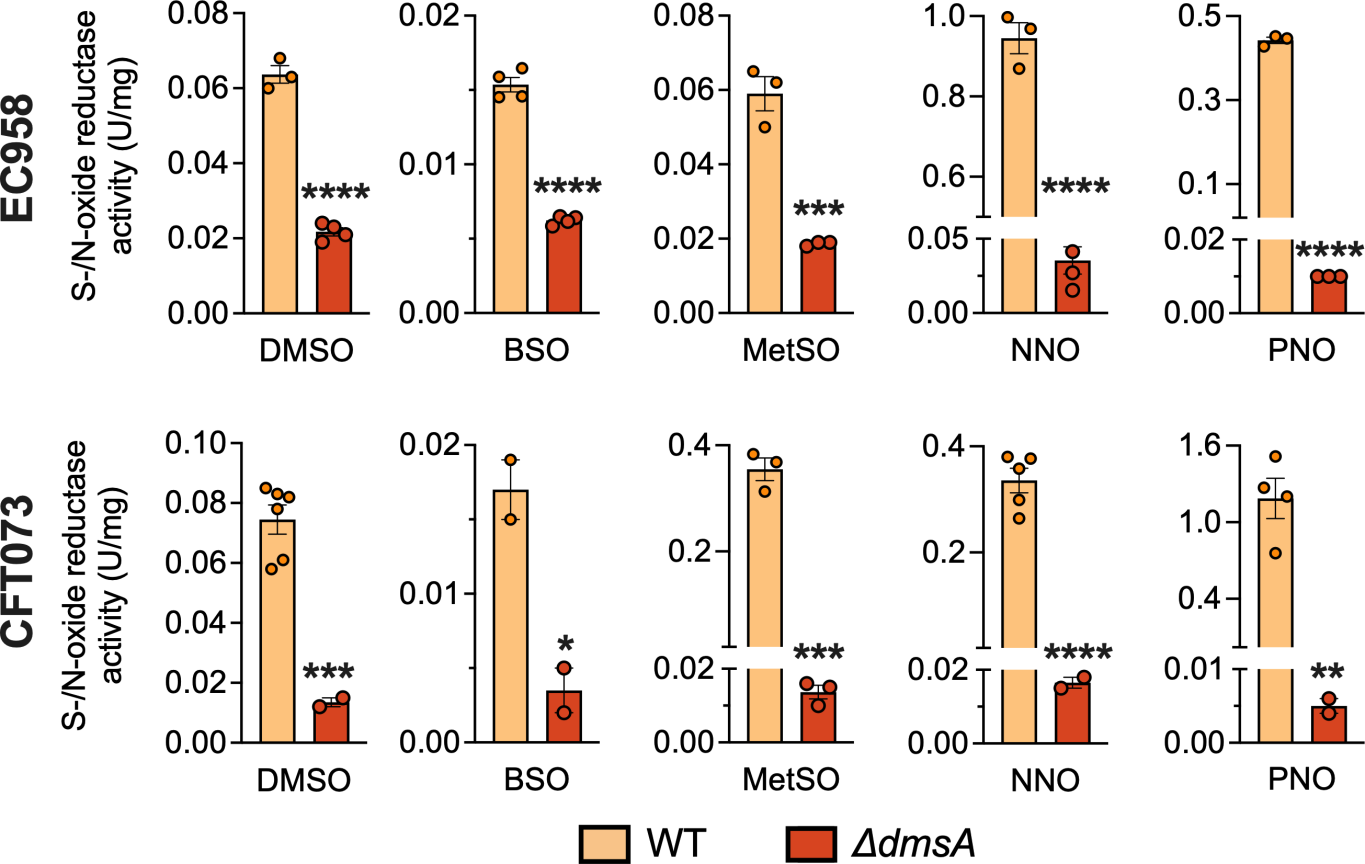
S-/N-oxide reductase activity in cell-free extracts from *E. coli* strains WT and ∆*dmsA* strains with biologically relevant S- and N-oxide molecules. S- and N-oxide reductase activity assays were performed using one of the following substrates: 10 mM dimethyl sulfoxide (DMSO), 5 mM dl*-*methionine sulfoxide (MetSO), 3 mM *S-*biotin-*S*-sulfoxide (BSO), 2.5 mM pyrimidine N-oxide (PNO), or 0.6 mM nicotinamide N-oxide (NNO). Representative data for several repeat experiments are shown; data points are technical replicates. Error bars represent the standard error of the mean. Two-tailed unpaired *t*-tests were performed to evaluate differences in enzyme activities: *P ≤ 0.05; **P ≤ 0.01; ***P ≤ 0.001; and ****P ≤ 0.0001. WT, wild-type.

The positive control, DMSO, worked as expected, with the EC958 and CFT073 ∆*dmsA* strain lysates showing 34% and 18% of WT level activity, which was very similar to values determined during mutant verification (Fig. 4). For all other substrates, activities in the ∆*dmsA* strain lysates were reduced by 60–98% (Fig. 4). The loss of activity was most pronounced for PNO and NNO, with activity reductions of 95–99%, while for DL-MetSO, activity reductions of 96% and 68% were observed for CFT073 and EC958, respectively (Fig. 4). Although catalytic parameters for each substrate could not be determined here due to the presence of several additional S- and N-oxide reductases in *E. coli* that could contribute to activity in cell lysates*,* and the purification of EcDmsABC was beyond the scope of the current work. The loss of activity with biologically relevant S- and N-oxide molecules in the ∆*dmsA* strains suggests that EcDmsABC contributes to the repair of oxidatively damaged biomolecules that can form as a result of the formation of reactive oxygen or chlorine species during interactions between *E. coli* and host cells. The activity profile also matches data collected for HiDmsABC (18), and further supports our proposal that DmsABC from *E. coli* and *H. influenzae* might have similar physiological functions. HiDmsABC converted both PNO and NNO with low micromolar *K*_M_ values (18), which, together with the strong attenuation of activity with PNO and NNO in the *E. coli* ∆*dmsA* strain lysates, suggests that *in vivo*, DmsABC might primarily convert N-oxide substrates.

### *E. coli dmsA* mutant strains show reduced survival during infection of T24 human bladder tissue cells

As the results so far suggest that EcDmsABC could have a role in host-pathogen interactions, we then carried out infection assays using T24 human bladder tissue cells and 2 and 4 h infection times.

At 2 h post-infection, CFU/mL for the EC958 ∆*dmsA* strain was comparable to the WT strain, while the CFT073 ∆*dmsA* strain displayed a 9-fold reduction (*P* < 0.0001) of the total host-associated bacteria (Fig. 3B). By 4 h post-infection, the EC958 ∆*dmsA* and CFT073 ∆*dmsA* strains showed 7-fold (*P* < 0.0001) and 75-fold (*P* < 0.0004) reductions in host-associated bacterial count, respectively (Fig. 3B).

We also attempted to assess levels of intracellular colonization at 4 h post-infection; however, none of the changes observed were statistically significant (Fig. S2). The EC958 and CFT073 WT strains showed comparable intracellular bacterial counts, and while the EC958 ∆*dmsA* strain showed 60% less viable cell, no intracellular bacteria were detected for the CFT073 ∆*dmsA* strain (Fig. S2). The data indicate that *E. coli* DmsABC is required for survival in contact with the T24 cells and may affect intracellular survival, which is consistent with our previous findings for DmsABC from *H. influenzae* (5).

### Additional Mo-containing S-/N-oxide reductases could contribute to host-pathogen interactions

As we were only able to identify a comparatively mild infection-related phenotype in UPEC ∆*dmsA* strains, we hypothesized that in *E. coli*, one or more of the six additional S-/N-oxide reductases that are functionally similar to DmsABC might compensate for its absence. Enzymes related to DmsABC include YnfE and YnfF, which are paralogues of DmsA and are part of the YnfEFGH selenate reductase (17, 47) and several S-/N-oxide reductases of the Dor-/Tor-type, which mostly consist of a periplasmic catalytic subunit and a membrane-bound cytochrome subunit (34). These include the TMAO reductase TorA that can only convert N-oxide substrates, the less characterized TorZ enzyme that has also been proposed to reduce TMAO as its major substrate (48–50), and the cytoplasmic BSO reductase, BisC. Finally, the MsrP peptide MetSO reductase, which has been shown to repair oxidative damage to protein-bound methionine residues, represents a third type of Mo-containing S-/N-oxide reductase found in *E. coli* (51). Except for BisC, these S-/N-oxide reductases are all located in the bacterial periplasm, where oxidative damage caused by extracellular HOCl would occur first (33, 34), and interestingly, the expression of *msrP* has already been shown to be induced by HOCl exposure (51, 52).

**Fig 5:**
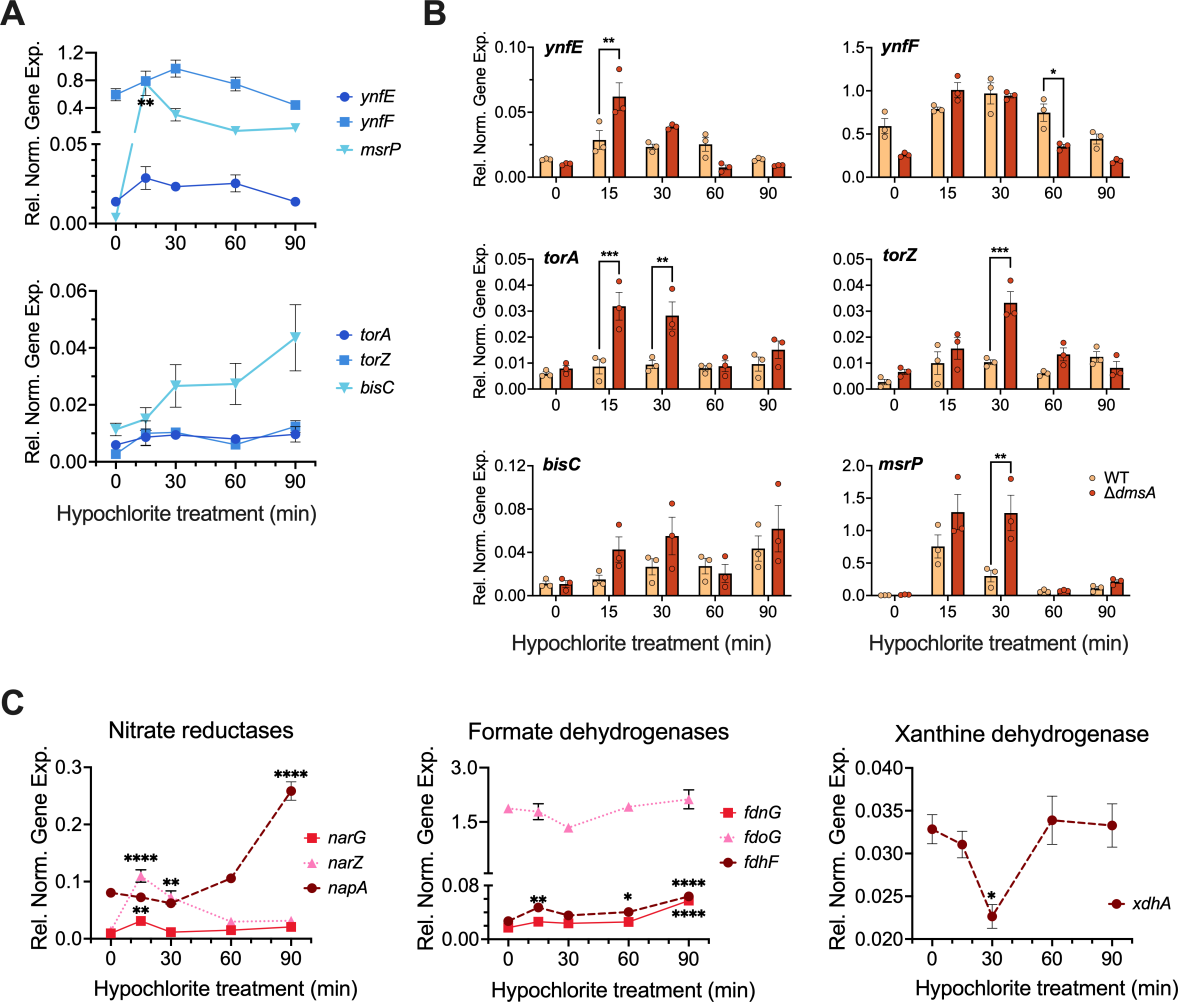
Expression of *E. coli* Mo enzymes in response to challenge of microaerobic cultures with 50 µM HOCl. Panel (A): Expression of S- and N-oxide reductase encoding genes in the EC958 WT strain following HOCl exposure. Panel (B): Comparison of S-/N-oxide reductase gene expression levels in EC958 WT and ∆*dmsA* strains following HOCl exposure. Panel (C): Expression levels of other Mo enzymes in EC958 WT strain following HOCl exposure. All gene expression data were normalized using expression of the *gyrA* gene. Three individual experiments were carried out; a representative data set is shown. Data points represent technical replicates; error bars represent the standard error of the mean. One-way ANOVA was performed for Panels (A and C). For Panel (B), two-way ANOVA was performed with Tukey’s correction for multiple comparisons: *P ≤ 0.05; **P ≤ 0.01; ***P ≤0.001; and ****P ≤ 0.0001. ANOVA, analysis of variance; HOCl, hypochlorite; WT, wild-type.

We hypothesized that the expression of an enzyme that could compensate for the function of DmsABC would also have to be induced by HOCl. Therefore, we tested the expression of *E. coli* S-/N-oxide reductase genes in MA cultures treated with 50 µM HOCl for up to 90 min. Prior to HOCl treatment, all S-/N-oxide reductase genes, except for *ynfF*, showed low levels of expression (Fig. 5A). Following exposure to HOCl, the expression of four enzymes, *ynfE*, *torA*, *torZ, and msrP* increased within 15 min by between 2-fold and 200-fold (Fig. 5A). Consistent with previous reports, *msrP* expression initially increased 200-fold (51–53), but decreased between 15 and 60 min, before remaining stable at 29-fold induction until the end of the experiment. Expression levels of *ynfE* and *ynfF* both showed an approximately 2-fold maximal increase that returned to pre-exposure levels by 90 min post-exposure. Expression of *torA* and *torZ* increased 2- and 4-fold by 15 min post-exposure, respectively, and remained elevated for the duration of the experiment. By contrast, the expression of *bisC* continued to increase after an initial 2-fold induction to a maximal 4-fold induction at 90 min post-exposure.

Next, we compared S-/N-oxide reductase gene expression levels in HOCl-treated EC958 WT and ∆*dmsA* cultures. With the exception of *ynfF*, where expression levels remained close to WT levels, 15 min after HOCl exposure, expression of the S-/N-oxide reductase genes was elevated in the EC958 ∆*dmsA* strain compared to the WT strain (Fig. 5B). Statistically significant increases were observed for *msrP*, *ynfE*, *torA,* and *torZ* between 15 and 30 min of HOCl exposure. From 60 min post-challenge, the expression of all genes was comparable to the WT strain (Fig. 5B). This compensatory overexpression of S-/N-oxide reductase genes could mask the physiological effects of a loss of DmsABC in the EC958 ∆*dmsA* strain. Additionally, the significant level of redundancy in S-/N-oxide damage repair enzymes in *E. coli* and the common gene regulation patterns in response to HOCl treatment highlight the importance of this function for the survival of the bacteria.

### Oxidative stress triggers the expression of the majority of *E. coli* Mo enzymes

As HOCl-based induction was surprisingly widespread among Mo-containing S-/N-oxide reductases, we then tested whether HOCl induction is also common for other Mo enzymes found in *E. coli* such as formate dehydrogenases, nitrate reductases, and xanthine dehydrogenases (Fig. 5C). Expression of genes encoding the two membrane-bound nitrate reductases, *narZ* and *narG*, peaked at 15 min post-exposure with 3- and 8-fold upregulation, respectively, before returning to pre-exposure levels. In contrast, the expression of the gene for the periplasmic nitrate reductases, *napA*, increased from 30 min after HOCl exposure until the end of the experiment (~3-fold induction) (Fig. 5C). Interestingly, two genes, *fdoG* and *xdhA*, encoding a formate dehydrogenase and a xanthine dehydrogenase, respectively, showed a reduction in expression that peaked at 30 min post-exposure, before returning to pre-treatment levels by 90 min. Expression of the FdnG and FdhF formate dehydrogenases was essentially unchanged in response to HOCl exposure.

**Fig 6:**
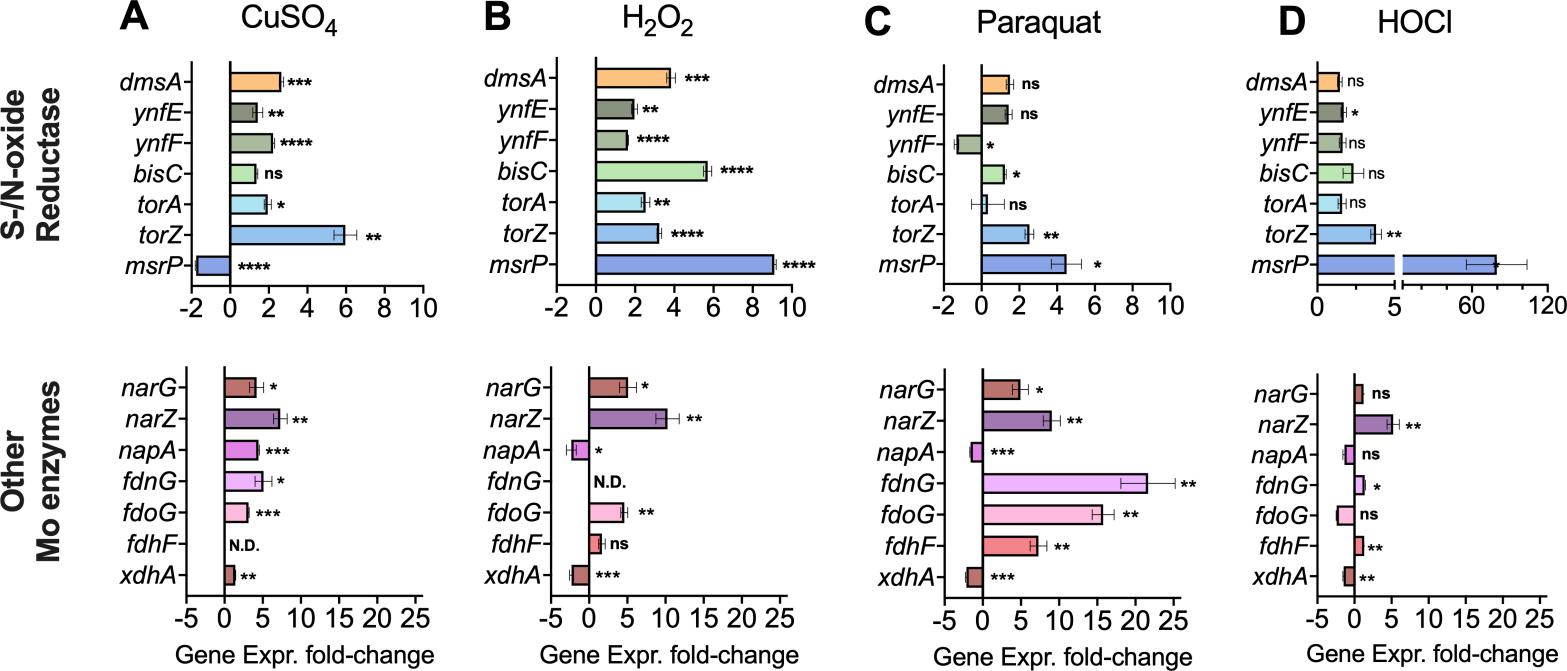
Expression of *E. coli* genes encoding Mo enzymes in response to exposure to oxidizing stressors. Panel (A): 150 µM copper sulfate, Panel (B): 10 mM hydrogen peroxide, Panel (C): 1 mM paraquat, and Panel (D): 50 µM HOCl. Data are shown as fold changes relative to the untreated EC958 WT strain. Exposure time was 30 min at mid-log phase, and all cultures were grown on M9 glucose. Fold changes are relative to the untreated sample. Error bars represent the standard error of the mean. Two-tailed unpaired *t*-tests were performed; underlying data are shown in Fig. S3: ns, not significant; *P < 0.05; **P < 0.01; ***P < 0.001; and ****P < 0.0001. HOCl, hypochlorite; WT, wild-type.

As HOCl treatment affected transcription of several key groups of Mo-enzyme encoding genes tested here and HOCl represents only one type of stress encountered by bacteria in the host environment, we expanded the investigation to include other oxidizing stressors that can occur in the host environment, namely hydrogen peroxide (H_2_O_2_), copper sulfate, which causes oxidative stress (54), and the superoxide-producer, paraquat. Exposure to these stressors for 30 min significantly changed the expression levels of the majority of genes, and in many cases, several stressors induced the expression of a particular gene (Fig. 6; Fig. S4). For S-/N-oxide reductase genes, H_2_O_2_ was the most potent inducer, leading to 2- to 9-fold increases in gene expression, with *msrP*, *bisC,* and *dmsA* being most strongly upregulated (Fig. 6). Copper stress is common at sites of infection (55) and increased expression of *torZ* (6-fold) and *dmsA* (3-fold) but not *msrP*, while paraquat treatment increased *msrP* expression 5-fold, followed by *torZ* expression that was 3-fold elevated. For Mo-enzymes other than S-/N-oxide reductases, formate dehydrogenase gene expression was induced between 7- and 22-fold by paraquat stress, and ~5-fold by CuSO_4_. However, only *fdoG* expression was also increased in the presence of H_2_O_2_. All nitrate reductases (*narG*, *narZ,* and *napA)* showed increased expression in the presence of CuSO_4_ stress. NarG and *narZ* expression also increased in the presence of paraquat and H_2_O_2_ as well as HOCl. Expression of the xanthine dehydrogenase, *xdhA*, increased in the presence of copper, but decreased for all other stressors (Fig. 6).

Our results demonstrate that the majority of mononuclear Mo enzymes in *E. coli* show gene expression changes following exposure to reactive chlorine and oxygen species as well as metal-induced stressors. While an association of MsrP and BisC with oxidative stress and infection processes has been previously documented (7, 52, 56), the data presented here suggest that the involvement of Mo enzymes in combating oxidative stress and supporting host-pathogen interactions is much more widespread than previously thought.

### DmsA in host-associated bacteria is phylogenetically distinct from DmsA in environmental bacteria

**Fig 7:**
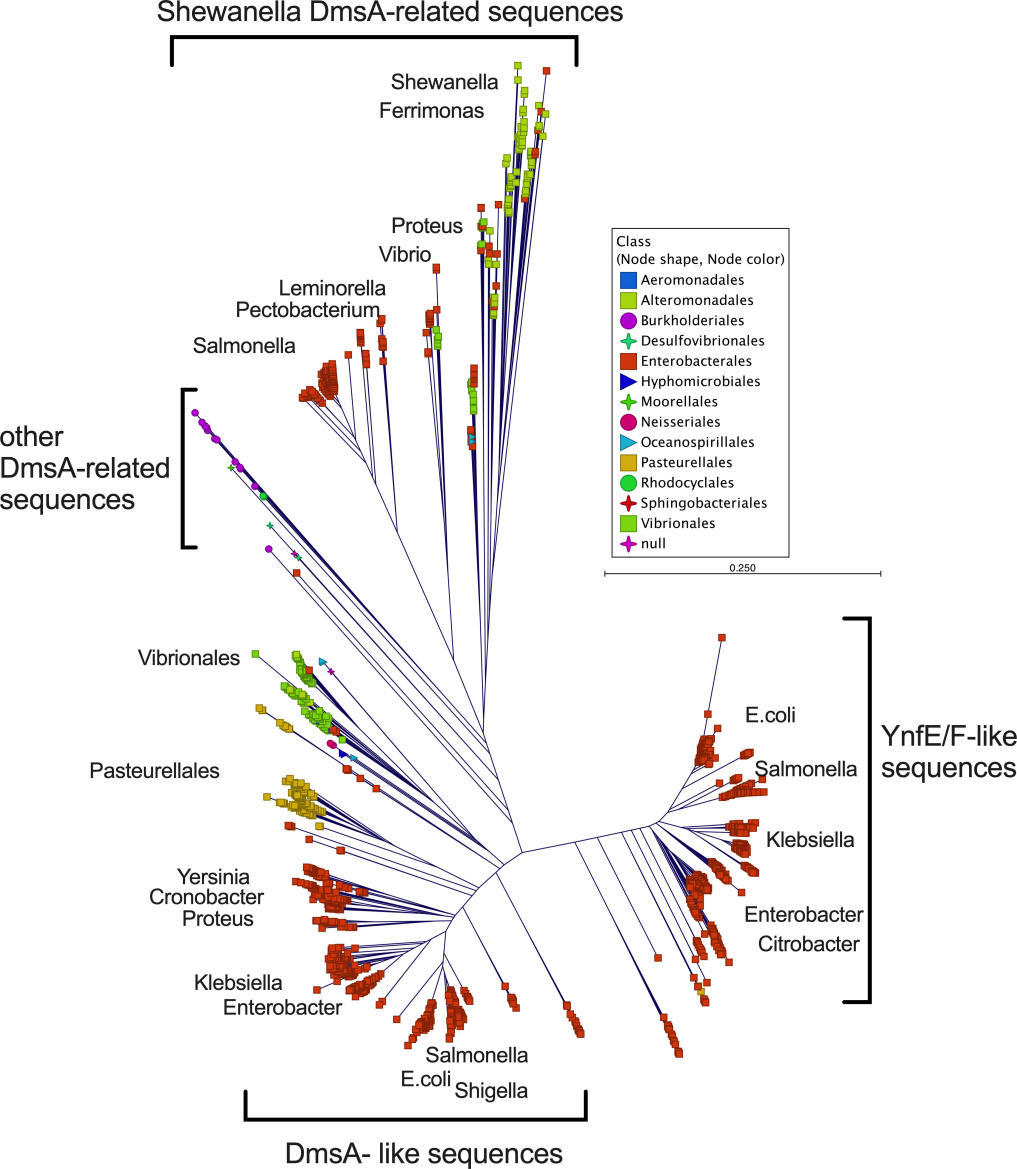
Phylogenetic analysis of DmsA-like protein sequences. Sequences were aligned, and the neighbour-joining tree constructed in CLC Genomics Workbench v24 (QIAGEN). Taxonomic details were derived from the NCBI Taxonomy database and overlayed as metadata.

Our data show that EcDmsABC likely has a primary role in protecting *E. coli* from oxidative damage rather than primarily supporting AN respiration with DMSO. However, EcDmsABC-related enzymes are also present in environmental bacteria such as *Shewanella* spp. that have been shown to use DmsABC to generate energy from environmentally available DMSO (57, 58). To better understand the relationships between these enzymes, we conducted a phylogenetic analysis of DmsA sequences that were identified using BLASTP searches with the DmsA sequences from *E. coli*, *H. influenzae,* and *Shewanella oneidensis*. After removal of duplicates and partial sequences, 6,498 sequences were used to generate a neighbor-joining tree (Fig. 7). Of these 6,498 sequences, 99.89% belonged to the phylum Pseudomonadota. Of the Pseudomonadota sequences, 99.54% originated from Gamma-Proteobacteria, and these were dominated by Enterobacterales (includes *E. coli*, 90.18%), followed by Vibrionales (4.2%), Pasteurellales (4.03%, including *H. influenzae*), and Alteromonadales (1.46%) to which *Shewanella* belongs. The remaining sequences originated from Beta-Proteobacteria (0.28%), as well as small numbers of Alpha-Proteobacteria. The 0.11% of non-Pseudomonadota sequences belonged to the phyla Bacillota, Thermodesulfobacteriota, Chloroflexota and Bacteroideta. The phylogenetic analyses revealed three major DmsA sequence groups, the *E. coli* DmsA-like sequences, *Shewanella* DmsA-like sequences, and Ynf-like sequences, each of which was composed of a range of often genus- or class-specific sequence groups (Fig. 7). Interestingly, the *Shewanella* DmsA-like sequences contained a range of sequences that originate from different Enterobacterales, and the majority of non-Pseudomonadota sequences grouped together between the *E. coli*- and *Shewanella* DmsA-like sequences.

As the sequences retrieved from the nr (non-redundant) database can be skewed due to the dissimilar numbers of available genomes for different genera, we also retrieved sequences from the Refseq_select database at NCBI using the same method as above. Here, we additionally filtered candidate sequences for the presence of the DmsA-specific Mo cofactor conserved domain (cd02770). In total, 1,221 sequences were retained, 71.42% of these belonged to the Pseudomonadota, and unexpectedly, 17.61% and 7.37% were Terrabacteria (former ‘gram-positive’ bacteria), from the Actinomycetota and Bacillota phyla. 1.64% and 1.15% of sequences belonged to genera from the Thermodesulfobacteriota and Campylobacterota, while representatives of the Deferribacterota, Spirochaeta, Chloroflexota, and Bacteroideta had abundances between 0.33% and 0.08% (Fig. S5). While this analysis confirmed that enzymes related to DmsABC occur mostly in phyla that contain Proteobacterial orders (Pseudomonadota and Campylobacterota), our analysis also revealed the existence of related enzymes in a comparatively large group of ‘gram-positive’ bacteria, and, in smaller numbers, in other bacterial phyla. Interestingly, while the studied representatives of DmsABC and related enzymes all have catalytic subunits located in the bacterial periplasm, of the sequences analyzed here, a small number of sequences (4.34% from both Pseudomonadota and Actinomycetota) were predicted not to contain the typical TAT-export signal that was present in ~94% of sequences, indicating further diversity of DmsA-related enzymes.

In summary, DmsA-like enzymes are widespread in the kingdom Bacteria, but there are a number of open questions, including whether DmsA-like sequences in the ‘Shewanella-group’ that originate from Enterobacterales could have a primary role in AN respiration rather than in oxidative stress mitigation, and what the physiological role of DmsA-like sequences in bacterial species from outside the Pseudomonadota and with non-periplasmic locations could be.

## DISCUSSION

The *E. coli* DmsABC S-/N-oxide reductase was one of the first bacterial Mo enzymes to be characterized in depth (10, 11) and has long been regarded as an enzyme with a primary function in AN energy generation. However, DMSO, the proposed main substrate of this enzyme during AN respiration, does not affect *dmsABC* gene expression (19).

Here, we have provided the first evidence that EcDmsABC has a role in host-pathogen interactions and associated stress responses, as not only known host-produced stressors, such as HOCl and H_2_O_2_ but also copper sulfate exposure increased *dmsA* expression (Fig. 1B, 5 and 6). Similar to what has been reported for an *H. influenzae* ∆*dmsA* strain (5), the *E. coli* ∆*dmsA* strain showed a reduced ability to adhere to human epithelial cells during infection (Fig. 3B). However, in *H. influenzae*, DmsABC is either the only or one of only two S-/N-oxide reductases (5, 18, 59), while six additional Mo-containing S-/N-oxide reductases are encoded in *E. coli* genomes. In our experiments, the expression of all genes encoding *E. coli* S-/N-oxide reductases was induced by exposure to different oxidative stressors, and several S-/N-oxide reductase genes including *torZ*, *msrP*, and *ynfE* showed increased expression in the *E. coli* ∆*dmsA* strain following exposure to HOCl, suggesting functional redundancy.

The upregulation of known S- and N-oxide reductases in the *E. coli* ∆*dmsA* strain following HOCl exposure correlates with our investigation of possible natural substrates for DmsABC. In addition to MetSO that had been previously identified as a substrate of DmsABC (5, 18, 46), EcDmsABC converted two N-oxides formed on precursors for NAD and pyrimidine, NNO and PNO, with high activities (Fig. 3), both of which were not previously described as substrates for EcDmsABC. While DMSO was also converted with good activities and our Δ*dmsA* strains were unable to grow by AN DMSO respiration, it is unlikely to play a major role as a DmsABC substrate during host-bacteria interactions, as there is only indirect evidence for the presence of DMSO in the gut lumen, and it is not found in human urinary tract epithelia (4). We have recently reported a very similar substrate profile for the closely related DmsABC from *H. influenzae* (DmsA: 73% aa sequence identity), which converted NNO, PNO, and MetSO with low micromolar *K*_M_ values (NNO: 32 ± 13 µM, PNO: 76 ± 17 µM, and MetSO: 35 ± 9 µM) that are within a physiologically relevant range (18). This confirms the functional conservation of DmsABC function in oxidative stress defense across bacterial species. More detailed enzymological characterization of the *E.coli* DmsABC substrate profile and comparisons to previous work that explored the DmsABC substrate range would require purified enzymes and are beyond the scope of the current investigation.

Functional redundancy of S-/N-oxide repair enzymes similar to what we demonstrated in this study has recently been shown to exist in *S. enterica* serovar Typhimurium, where a triple gene knockout of *dmsABC* and two additional genes, STM0964 and STM4305, that encode related enzymes, led to a reduction in host colonization (4). Some functional redundancy was also observed for the *H. influenzae* MtsZ and DmsABC S-/N-oxide reductases (18). In fact, functional redundancy has been shown to mask virulence-related phenotypes for a variety of cellular processes, including iron acquisition, phospholipid hydrolysis, and protein glycosylation, and has been documented in pathogenic bacteria such as *E. coli*, *Pseudomonas aeruginosa*, *Burkholderia pseudomallei*, and *Legionella pneumophila* (60–63). The significant level of S-/N-oxide reductase redundancy in *E. coli*, *Salmonella* sp., and even a species with a highly reduced genome size such as *H. influenzae* suggests that mitigation of S-/N-oxide stress is an essential function for in-host survival of host-associated bacteria.

Interestingly, our experiments on HOCl exposure of an *E. coli* ∆*dmsA* strain revealed increased gene expression of *msrP* (Fig. 5B), which encodes a peptide MetSO reductase with a role in repairing sulfoxide damage to proteins (51). MsrP maintains bacterial cell envelope integrity by reversing oxidative damage to methionine residues in a wide range of periplasmic proteins, including the SurA chaperone and the Pal lipoprotein (51). While it is not immediately obvious how an enzyme that uses damaged proteins as substrates would functionally replace DmsABC, which appears to function best with small molecule substrates (46), a similar observation has been made in *H. influenzae*, where expression of both DmsABC and MsrAB, an enzyme with a function equivalent to MsrP in repairing damaged outer membrane and periplasmic proteins was triggered by HOCl-exposure (18, 64). In *H. influenzae*, DmsABC and MsrAB form a novel, periplasmic stress-defense system (18), and the upregulation of *msrP* expression in the *E. coli* ∆*dmsA* strain could suggest that a similar S-/N-oxide repair system exists in *E. coli*.

Another unexpected finding was that expression of many *E. coli* Mo-enzymes was induced by oxidative stress agents such as HOCl, H_2_O_2_, copper sulfate, and paraquat. While MsrP had been previously demonstrated to be induced by HOCl (51, 52), for most other *E. coli* Mo-enzymes, a connection to oxidative stress responses has not been specifically documented so far. A question that then arises is which regulators are involved in the observed gene expression changes. To date, a link between stress-dependent expression of a Mo enzyme and a specific regulator has only been established for the *E. coli msrP*, which is regulated by two-component systems HprSR and CusSR (52, 56, 65). In *E. coli*, responses to peroxide and superoxide stress are mediated by OxyR and SoxSR; however, none of the Mo enzymes investigated here have been reported to belong to either regulon (66, 67). Several HOCl-responsive regulators, HypT, NemR, and RclR, have also been identified in *E. coli*, but regulon data are only available for RclR, and it does not include *dmsA* or any other Mo enzyme encoding genes (68–71). However, a recent study of RcrR, another HOCl-responsive repressor, reported transcriptomic data for *E. coli* CFT073 exposed to 2.5 mM HOCl for 15 min in a minimal salt medium, where five Mo enzyme complexes were upregulated following HOCl exposure (53). This included all components of the DmsABC and MsrP/Q sulfoxide reductases, as well as subunits of the FdnGHI formate dehydrogenase and the NarGHI nitrate reductase, which aligns with our results. Future work should investigate whether HOCl-dependent induction of Mo enzyme expression is mediated by any of these known systems, or may involve other, as yet unstudied regulators. Investigations into the relevant regulatory circuits may also reveal the molecular basis for the strain-specific differences in the magnitude of physiological changes that are evident in our data.

In conclusion, we have demonstrated here that, contrary to the long-held belief that DmsABC supports AN respiration with DMSO in *E. coli*, the physiological role of EcDmsABC appears to be in defense against a variety of oxidizing stressors, where the enzyme’s likely role is to restore the function of oxidatively damaged biomolecules such as methionine, nicotinamide, and pyrimidine. This is the first time that specific inducers of gene expression have been identified for EcDmsABC, and aligns the function of EcDmsABC with the emerging role of DmsABC as a determinant of successful host-pathogen interactions (4, 5, 17). Our data indicate that this type of function may be common in additional Mo enzymes, and the association between Mo enzyme function, bacterial virulence, and oxidative stress responses should be further investigated in the future. Finally, our phylogenetic analyses revealed a previously undocumented diversity in DmsABC-like enzymes that extends beyond the Pseudomonadota to two groups of ‘gram-positive’ bacteria, and also indicates evolutionary specialization of DmsABC-like enzymes to suit different physiological roles and contexts that will require further investigation.

## MATERIALS AND METHODS

### Bacterial strains and growth conditions

Details of bacterial strains and plasmids used in this study are shown in Tables S2 and S3. *E. coli* strains were cultured routinely at 37°C in liquid or solid LB medium (72), or liquid M9-glucose medium (73) with 200 rpm shaking. Liquid glycerol minimal medium was also used with supplementation of 70 mM DMSO or 40 mM fumarate as described in reference 44 with a reduced CaCl_2_ concentration. AN cultures were incubated statically in completely filled tubes for 16–18 h for LB medium or 40–42 h for glycerol medium. Kanamycin (Kan, 100 µg/mL), chloramphenicol (Cam, 30 µg/mL), gentamicin (Gen, 20 µg/mL), or ampicillin (Amp, 100 µg/mL) were added to the growth media when appropriate. Where required, media were supplemented with ferric citrate (10 µg/mL), ferrous sulfate (10 µg/mL), and sodium molybdate (1 mM).

For growth experiments, actively growing liquid bacterial cultures (at an OD_600_ of 0.6–0.7 in M9-glucose medium) were diluted to an OD_600_ of 0.05 and distributed into a 96-well U-bottom polystyrene microtiter plate (200 µL per well). OD_600_ values were collected every 30 min under MA (2.8% O_2_, 5% CO_2_, with 200 rpm shaking) or AN (0.3% O_2_, 5% CO_2_) conditions at 37°C using a CLARIOstar Multimode plate reader with an atmospheric control unit (BMG Labtech). To test the effect of HOCl on growth, the bacterial cultures were diluted to an OD_600_ of 0.1 and mixed with an equal volume of double-strength HOCl (freshly prepared in the growth medium, final conc: 15 and 20 µM) and incubated as above. Higher HOCl concentrations were used for LB medium because the high content of organic molecules that can react with HOCl reduces the effect of the compound on the bacterial cells (74). All growth rates and lag times were determined using the *GrowthRates* program (v4.41) (75).

### General molecular biology and biochemical methods

Standard methods were used throughout (72). All chemicals were purchased in analytical grade unless otherwise indicated. Plasmid isolation and PCR purification used GeneJET Plasmid Miniprep Kit and PCR Purification Kit (Thermo Fisher Scientific). Genomic DNA was isolated using DNAzol (Thermo Fisher Scientific). DNA concentrations were determined on a Nanodrop One (Thermo Fisher Scientific). PCR reactions were performed with Gotaq Green Master Mix (Promega) or Phusion Flash High-Fidelity PCR Master Mix (Thermo Fisher Scientific) as per the manufacturer’s instructions. Oligonucleotides were purchased from IDT (Table S4). Plasmid construction used the NEBuilder HiFi DNA Assembly Kit (NEB) as recommended by the manufacturer. Restriction enzymes were from Thermo Fisher Scientific and NEB. Ligation reactions were performed with the T4 ligase (NEB) in 10 µL reactions with a 3:1 insert:vector ratio, which were incubated at room temperature for 1 h and directly transformed into competent *E. coli* by heat shock (76). Sequencing reactions used BigDye v3.1 (Thermo Fisher Scientific) provided by the Genome Research Service (The University of Queensland, Australia). Cell-free extracts were generated using BugBuster Protein Extraction Reagent (Merck) at a 1:3 to 1:5 ratio of cell pellet weight (g) to reagent volume (mL) as per the manufacturer’s instructions; cell debris was removed from the cell extract by centrifugation at 21,300 × *g* at room temperature for 3 min.

### RNA isolation and quantitative real-time PCR (qRT-PCR)

MA bacterial cultures for RNA isolation (100 mL culture in a 250 mL flask) were grown to the mid-log phase at 37°C with shaking at 200 rpm and then exposed to stress-inducing agents. HOCl was added at final concentrations of 50 µM (M9) or 200–300 µM (LB), and 2 mL culture samples were collected before (time 0) and after 15, 30, 60, 90, and 120 min of HOCl exposure. Samples were preserved using RNAprotect Bacteria Reagent (QIAGEN). For cultures exposed to H_2_O_2_ (10 mM), copper sulfate (150 µM) or paraquat (1 mM) in M9 medium, RNA isolation samples were collected at time 0 and 30 min post-exposure. RNA isolation and purification used the RNAspin Mini kit (Cytiva), and genomic DNA was removed using the Turbo DNA-free Kit (Ambion). To confirm successful gDNA removal, PCR reactions with purified RNA as the template and primers CFT16SQP F and R (Table S4) were used. RNA was quantified using the Qubit RNA HS Assay (Thermo Fisher Scientific). First-strand cDNA synthesis with the SuperScript IV or Superscript IV VILO Master Mix (Thermo Fisher Scientific) used 500 ng of RNA and random hexamer primers (Thermo Fisher Scientific).

QuantiNova SYBR Green Master Mix (QIAGEN) was used to prepare qRT-PCR reactions (10 µL, final volume) with diluted cDNA (1:100) as template, and primer concentrations of 2 µM (Table S4). Data from qRT-PCR were analyzed as detailed in reference 5; PCR efficiencies were determined using LinReg (77). Gene expression levels were normalized against *gyrA* expression.

### Construction and complementation of *E. coli* ∆*dmsA* strains

The pBluEcdmsA-KO_Km plasmid, which carries the *dmsA* gene with an insertion-deletion mutation, was created using Gibson Assembly of four fragments: a pKD4-derived kanamycin-resistance cassette (1,478 bp, primers: UTI89_dmsA_KO_F and UTI89_dmsA_KO_R), two fragments of the *E. coli dmsA* gene (437 and 560 bp, primer pairs: dmsA_pBlu_GB_F, dmsAko_int_up_R, dmsA_pBlu_GB_R, and dmsAko_int_down_F, respectively), and a pBluescript II backbone (primers: pBlu_GB_F and pBlu_GB_R). Plasmid pBluEcdmsA-KO_Cm was generated by replacing the kanamycin-resistance cassette in pBluEcdmsA-KO_Km with a pKD3-derived, chloramphenicol-resistance cassette (1,019 bp, primers pKD3_CM3a and pKD3_CM4a, EcdmsA_int_KO_Cm_F and EcdmsA_int_KO_Cm_R). DNA fragments containing the disrupted *dmsA* (*dmsA*-KO_Km and *dmsA*-KO Cm) were PCR-amplified from pBluEcdmsA-KO_Km and pBluEcdmsA-KO_Cm, using primers EcdmsAKO_F and EcdmsAKO_R. Following DpnI treatment, these PCR products were used to replace the chromosomal copy of *dmsA* in *E. coli* CFT073 and EC958 WT strains using the lambda Red-mediated recombination system of the pKOBEG-G plasmid (42). Mutations were confirmed by colony PCR screens and Sanger sequencing.

Complementation was achieved by transformation of *E. coli* ∆*dmsA* strains with the pSU-EcdmsABC-G plasmid. Primers EC958_dmsA_compF_SacI and EC958_dmsA_compR_XmaI were used to amplify 4,440 bp of the EC958 *dmsABC* gene region including the *dmsA* promoter. The PCR product was cloned into pSU2718-G using the SacI and XmaI sites (43) (Table S4). Complementation of the CFT073 and EC958 ∆*dmsA^C^* strains was confirmed by colony PCR screens.

### Enzyme activity assays

S-/N-oxide reductase activity was determined using cell-free extracts in a Cary 60 spectrophotometer (Agilent Technologies) as detailed in reference 59. Reactions contained 20 mM Tris-Cl (pH 7.5) buffer containing 0.06 mM methyl viologen and one of the following substrates: 10 mM DMSO, 5 mM dl-MetSO, 3 mM *S-*biotin-*S*-sulfoxide (kindly provided by Professor Paul Bernhardt’s laboratory at The University of Queensland, Australia), 2.5 mM PNO, or 0.6 mM NNO. Assay mixtures were degassed with nitrogen, then freshly dissolved 0.1 M sodium dithionite was used to reduce the methyl viologen before addition of cell lysate or substrate to initiate the reaction. Absorbance changes were monitored at 600 nm. An extinction coefficient of 13.7 mM^−1^ cm^−1^ for methyl viologen was used to determine enzyme activities (78). Enzyme activities are given as μmoles of substrate reduced per min and mg of protein or U/mg present.

### HOCl susceptibility assays

Bactericidal assays were carried out using 0–50 µM HOCl essentially as previously described (5). Overnight cultures of EC958 and CFT073 strains in LB medium were harvested at 2,370 × *g* for 10 min at room temperature and washed three times in 1× phosphate-buffered saline (PBS). A total of 900 µL of bacterial culture containing approximately 2 × 10^8^ CFU/mL suspension were combined with a 10× stock solution of the stressor (5). Samples were incubated at room temperature with gentle rocking for 60 min, followed by immediate serial dilution in PBS (10^0^ to 10^−7^), spotting of 5 µL per dilution on LB agar, and determination of viable bacterial counts following overnight incubation.

### Tissue cell culture and infection assays

The human bladder cancer cell line T24 (kindly provided by Professor Mark Schembri at The University of Queensland) was cultured in modified McCoy’s 5A medium (Sigma-Aldrich) supplemented with 10% heat-inactivated fetal calf serum (Thermo Fisher Scientific), as previously reported (39–41). Infection assays were performed in 24-well plates as in reference 79. Briefly, each well was seeded with 1 × 10^5^ T24 cells and incubated overnight at 37°C with 5% CO_2_. Tissue cells were then infected at an MOI of (100:1) with freshly grown *E. coli* cultures diluted in supplemented McCoy’s 5A medium to a concentration of 5 × 10^7^ bacteria/mL. To determine the total T24-associated bacterial CFUs after a 2-h incubation at 37°C with 5% CO_2_, planktonic bacteria were removed, infected tissue cells were washed three times with 1× PBS, and lysed using 0.1% Triton X-100 (in PBS). CFU counts were determined by serial dilution in PBS (10^0^ to 10^−7^) and plating of 5 µL per dilution on LB agar. To determine total T24 associated or intracellular bacteria at 4 h post-infection, samples were washed (three times, 1× PBS) at 2 h post-infection before addition of either fresh supplemented McCoy’s medium (total cells) or supplemented McCoy’s medium containing either 1 µg/mL (CFT073) or 2 µg/mL (EC958) polymyxin B (intracellular bacteria determination). Cultures were incubated for an additional 2 h before CFU determination as described for the 2-h time point.

### Phylogenetic analyses

DmsA amino acid sequences (*E. coli*—WP_000850286.1; *H. influenzae*—WP_044364421.1; and *Shewanella oneidensis*—WP_011071631.1) were retrieved from the NCBI protein database. Homologous sequences were identified using BLASTP (nr) database; output: 5,000 per search), and outputs including taxonomic data retrieved from NCBI. The retrieved sequences were combined before duplicates and partial sequences (<700 amino acid length) were removed. A total of 6,489 unique sequences were aligned, and a neighbor-joining tree constructed using the Jukes-Cantor substitution model and 100 bootstrapping cycles using the CLC Genomics Workbench Alignment and Phylogeny tools (QIAGEN). Taxonomic metadata based on the NCBI taxonomy database were overlayed onto the tree using the CLC Genomics Workbench. Additional phylogenetic analyses used the same input sequences and BLASTP searches using the NCBI REFseq_select database. Output sequences were combined, duplicates removed, and further filtered for the presence of the MopB_DmsA_EC conserved domain, cd02770. This resulted in 1,221 unique sequences. These sequences were analyzed as described above.

### Statistical analyses

Statistical analyses were carried out using Prism 9 (v.9.5.1; GraphPad Software Inc., USA). Two-tailed unpaired *t*-tests (gene expression level data and enzyme activity data), one-way analysis of variance (ANOVA) (enzyme activity data, tissue cell infection data, gene expression level data, and HOCl sensitivity data) or two-way ANOVA (growth lag data and gene expression level data) tests were carried out with Tukey’s multiple comparisons test. A *P* value ≤0.05 was considered statistically significant.

## SUPPLEMENTAL MATERIAL

Supplemental figures and tables jb.00463-24-s0001.docx Figures S1 to S4 and Tables S1 to S4.

ASM does not own the copyrights to Supplemental Material that may be linked to, or accessed through, an article. The authors have granted ASM a non-exclusive, world-wide license to publish the Supplemental Material files. Please contact the corresponding author directly for reuse.
