# Supplementary material for: Beyond anaerobic respiration—new physiological roles for DmsABC and other S-/N-oxide reductases in Escherichia coli
Source: J Bacteriol. 2025 Mar 31;207(5):e00463-24. doi: 10.1128/jb.00463-24 (PMC12096835; doi:10.1128/jb.00463-24)
Supplement: Supplemental figures and tables — Figures S1 to S4 and Tables S1 to S4. [file jb.00463-24-s0001.docx]

**Beyond anaerobic respiration – new physiological roles for DmsABC and other S-/N-oxide reductases in *Escherichia coli***

Qifeng Zhong^1^, Marufa Nasreen^1^, Ruizhe Yang^1^, Michel Struwe^1^, Bostjan Kobe^1,2^, Ulrike Kappler^1^

^1^School of Chemistry and Molecular Biosciences, The University of Queensland, St. Lucia, QLD 4072, Australia

^2^Institute for Molecular Bioscience, The University of Queensland, St. Lucia, QLD 4072, Australia

**Supplementary Materials**

[Figure S1. **Relative normalised gene expression of *dmsA* in uropathogenic *Escherichia coli* EC958 WT strain.** 2](#_Toc177331548)

[Figure S2. **Invasion phenotypes of UPEC EC958 and CFT073 strains during interactions with human tissue cell line.** 3](#_Toc177331549)

[Figure S3. **Relative normalized gene expression of *E. coli* Mo enzymes in response to oxidising stressors.** 4](#_Toc177331550)

[Figure S4. **Phylogenetic tree of DmsA-like sequences identified using the Refseq Select Protein database.** 5](#_Toc177331551)

[Table S1. Growth rates of UPEC strains EC958 and CFT073 following HOCl treatment 7](#_Toc177332063)

[Table S2. Plasmids used in this study 8](#_Toc177332064)

[Table S3. strains used in this study 9](#_Toc177332065)

[Table S4. Oligonucleotide primers utilized in this study 10](#_Toc177332066)

Figure S1. **Relative normalized gene expression of *dmsA* in uropathogenic *Escherichia coli* EC958 WT strain.** The culture was treated with 50 µM HOCl in M9-glucose minimal medium over a 90-min time course under microaerobic conditions. Data were normalized using the expression level of the *gyrA* gene. Data points represent technical replicates. Error bars represent the standard error of the mean. One-way ANOVA with Tukey’s multiple comparison correction was performed, statistical significance against the untreated sample is shown: *, *p*<0.05; ****, *p<0*.0001.

Figure S2. **Invasion phenotypes of UPEC EC958 and CFT073 strains during interactions with human tissue cell line.** Bacterial cultures were incubated with T24 human bladder epithelial cell lines and bacterial counts were measured at 4 h post-infection. Data points represent biological replicates and error bars represent the standard error of the mean. ND – not detected. One-Way ANOVA with Tukey’s multiple comparison correction was performed, statistical significance against the WT is shown: **, *p*<0.003.

Figure S3. **Relative normalized gene expression of *E. coli* Mo enzymes in response to oxidizing stressors.** Bacteria were cultured to mid-log phase in M9-glucose. Hydrogen peroxide (10 mM), copper sulfate (150 µM), paraquat (1 mM), or M9 medium in the (untreated control) were added and cultures incubated at 37°C for 30 min before sampling. Fold-change data are shown in Figure 6. Data points represent technical replicates. Error bars represent the standard error of the mean. Two-tailed unpaired t-tests were performed: *, *p*<0.05; **, *p<*0.01; ***, *p*<0.001; ****, *p*<0.0001. ND – not detected.


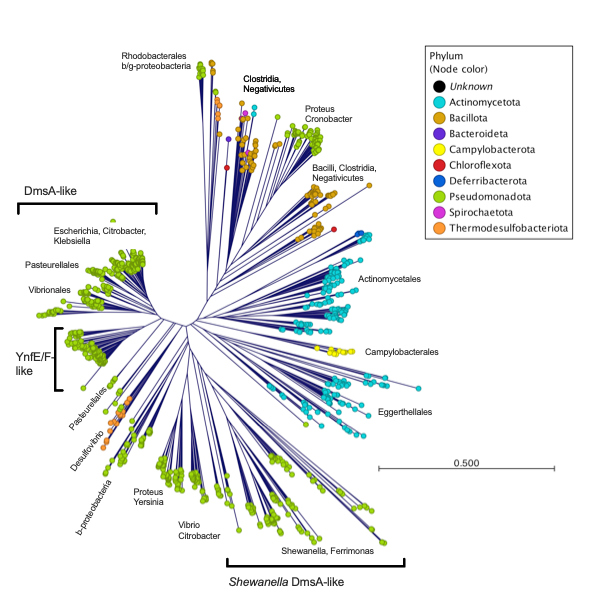


Figure S4. **Phylogenetic tree of DmsA-like sequences identified using the Refseq Select Protein database.** Sequences were aligned and the Neighbour-Joining tree constructed in CLC Genomics Workbench v24 (QIAGEN). Taxonomic details were derived from the NCBI Taxonomy database and overlayed as metadata.

Table S1. Growth rates of UPEC strains EC958 and CFT073 following HOCl treatment

Note: Bacterial cultures were incubated in M9-glucose medium under anaerobic or microaerobic conditions, and exposed to 0, 15 or 20 µM HOCl at the start of the incubation. CFT073 strains did not grow following treatment with 20 µM HOCl. Three independent experiments were conducted, and representative data from one experiment is shown. Values are shown as (mean±standard error of mean). Values were calculated using the *GrowthRates* program (v4.41) [73].

-, not determined.

Table S2. Plasmids used in this study

| **Plasmid** | **Relevant features** | **References** |
| --- | --- | --- |
| pBluescript II SK | SK (+), Amp^R^, *lacZ* | Stratagene |
| pSU2718-G | pACYC184, *trc* promoter, Gen^R^ | [43] |
| pKOBEG-G | lambda Red genes (γ, β, and *exo*), Gen^R^ | [39] |
| pBluEcdmsA-KO_Cm | pBluescript II derivative, *dmsA*:*cam* (with *dmsA* derived from *E. coli strain* UTI89), Cam^R^, Amp^R^ | This study |
| pBluEcdmsA-KO_Km | pBluescript II derivative, *dmsA*:*kan* (with *dmsA* derived from *E. coli strain* UTI89), Kan^R^, Amp^R^ | This study |
| pSUEcDmsABC-G | pSU2718-G derivative, Gen^R^ , *dmsABC* (from *E. coli* strain EC958) and a native *dms* promoter | This study |

Abbreviations: Amp – Ampicillin; Gen – Gentamicin; Cam – Chloramphenicol; Kan – Kanamycin.

Table S33. Bacterial strains used in this study

| ***E. coli* Strains** | **Relevant features** | **References** |
| --- | --- | --- |
| DH5alpha | *E. coli* cloning strain,  *Δ(argF-lac)169, φ80dlacZ58(M15), ΔphoA8, glnX44(AS), deoR481, rfbC1, gyrA96(NalR), recA1, endA1, thiE1* and *hsdR17* | Thermo Fisher Scientific |
| CFT073 | Wild-type UPEC reference strain | [40] |
| EC958 | Wild-type UPEC reference strain; Kan^R^, Amp^R^ | [39] |
| CFT073 *∆dmsA* | CFT073, *dmsA::kan,* Kan^R^ | This study |
| EC958 *∆dmsA* | EC958, *dmsA::cam,* Cam^R^ | This study |
| CFT073 *∆dmsA* comp | CFT073 *∆dmsA* harbouring pSU2718-EcdmsABC-G*,* Gen^R^ | This study |
| EC958 *∆dmsA* comp | EC958 ∆*dmsA* harbouring pSU2718-EcdmsABC-G*,* Gen^R^ | This study |

Abbreviations: UPEC – uropathogenic *E. coli;* Kan – Kanamycin; Amp – Ampicilin; Cam – Chloramphenicol; Gen – Gentamicin.

Table S44. Oligonucleotide primers utilized in this study

| **Primer Name** | **Primer sequence (5'-3')** |
| --- | --- |
| **qPCR primers** | |
| CFTbisCQPF | AAAACGTGCATAACCGCCCTTCTGC |
| CFTbisCQPR | GATGAAGCAAGTGGTGCCGCCA |
| CFTdmsAQPF | TATACCTACGGCGGCTGGGCAGAT |
| CFTdmsAQPR | TCATTCGCGTTTCGCCAGGGTT |
| CFTfdhFQPF | GTGAAGAACTGCGCCAGACCGT |
| CFTfdhFQPR | TTCAGTGGCCTTGCCGCGAT |
| CFTfdnGQPF | GAAGACGGTCTGTTCAGCGGCTAC |
| CFTfdnGQPR | CATCGCGTTTCGCATAGCCG |
| CFTfdoGQPF | GGAAGTGCTCGGTCAGACGATAGGT |
| CFTfdoGQPR | AATCCGGCGGCGCGTATCTT |
| CFTgyrAQPF | CGGCGCTGTTAGCGGTGATTTC |
| CFTgyrAQPR | AGCGCCGATCGTCTGATGGAAG |
| CFTnapAQPF | TTCGGTCCGGCCTGCATGTT |
| CFTnapAQPR | AAATCGGTCTGCACGCGGTG |
| CFTnarGQPF | CCGCAAACCTGGGGTGAGCAAACT |
| CFTnarGQPR | TACGCGTCTGCGGCACGTTT |
| CFTnarZQPF | TTCATAGGTAGCAGGGTAGTCGCGC |
| CFTnarZQPR | TGAAGTGCTCGACTGGCGCA |
| CFTtorZQPF | AAATCGTTGCGCGCTTCAAATTGC |
| CFTtorZQPR | TTGAGCGCAATGACCTGACGATGA |
| CFTxhdAQPF | AACTTGGCGGCATTCCGGTGA |
| CFTxhdAQPR | ACGCCCATTTGCCCGTCAAT |
| CFT16SQPF | GCCACACTGGAACTGAGACACGG |
| CFT16SQPR | ATACACGCGGCATGGCTGCA |
| UPECtorAQPFv2 | TGGGGCTCCGATTTGCTGAA |
| UPECtorAQPRv2 | CGGCGGCGACTTTCGC |
| ***dmsA* knockout construction** | |
| pBlu_GB_F | TGCAGCCCGGGGGATCCA |
| pBlu_GB_R | GCTTATCGATACCGTCGAC |
| dmsA_pBlu_GB_F | GGTCGACGGTATCGATAAGCAAAACGAAAATCCCTGATG |
| dmsA_pBlu_GB_R | AGTGGATCCCCCGGGCTGCACACCTTTTCAACCTGAAC |
| dmsAko_int_down_F | ACGTTGCTTATAAAGCTTTCCGTGAA |
| dmsAko_int_up_R | GATGTCGTAGGCTTCTTCCCAGC |
| UTI89_dmsA_KO_F | GGTGAAGGCAAATTCGAGCGCATTAGCTGGGAAGAAGCCTACGACATCATTGTGTAGGCTGGAGCTGCTTC |
| UTI89_dmsA_KO_R | GTCAGTGGATTTGCCTGCGGATCTTCACGGAAAGCTTTATAAGCAACGTGCATATGAATATCCTCCTTAG |
| EcdmsAintKo-CmF | CCTACACAATCGCTCAAGACTTCACGGAAAGCTTTATAAGCAACGT |
| EcdmsAintKo-CmR | GGAATAGGAACTAAGGAGGAGCTGGGAAGAAGCCTACGACATC |
| pKD3_CM3a | TCCTCCTTAGTTCCTATTCC |
| pKD3_CM4a | GTCTTGAGCGATTGTGTAGG |
| **dmsA-KO complementation** | |
| EC958_dmsA_compF_SacI | AAAAGAGCTCGCCACACACTTTATGATGAATGG |
| EC958_dmsA_compR_XmaI | AAAACCCGGGTACTCCTTACACCTTTTCAACCTG |
| **Screening of gene knockout and complemented mutant strains** | |
| EcdmsA_seq_S1 | TGCGCGGGAAAGATAAGCT |
| EcdmsA_seq_S2 | GTGCTGAAAGCAGCTTGCC |
| EcdmsC_seq_AS | GATCATCAGCGGCCATTCATGC |
| EcdmsC_seq_S | TGCTGATGGGCATTGGCT |
| EcdmsAKO_F | AAAACGAAAATCCCTGATGCGG |
| EcdmsAKO_R | CACCTTTTCAACCTGAACAAGGTT |
